# Organization of neuropeptide systems in the human brain

**DOI:** 10.1038/s41593-026-02236-w

**Published:** 2026-03-17

**Authors:** Eric G. Ceballos, Asa Farahani, Zhen-Qi Liu, Filip Milisav, Justine Y. Hansen, Alain Dagher, Bratislav Misic

**Affiliations:** Montréal Neurological Institute, McGill University, Montréal, Quebec Canada

**Keywords:** Neuroscience, Computational neuroscience

## Abstract

Neuropeptides are functionally diverse signaling molecules in the brain, regulating a wide range of basal bodily and cognitive processes. Despite their importance, the distribution and function of neuropeptides in the human brain remains underexplored. Here we comprehensively map the organization of human whole-brain neuropeptide receptors across multiple levels of description, including molecular and cellular embedding, mesoscale connectivity and macroscale cognitive specialization. Using gene transcription as a proxy, we reconstruct a topographical cortical and subcortical atlas of 38 neuropeptide receptors across 14 different neuropeptide families. We find that most neuropeptide receptors are highly expressed either in the cortex or subcortex, delineating an anatomical cortical–subcortical gradient. Mapping neuropeptide receptors onto hypothalamic nuclei, we demonstrate that neuropeptide receptor gene expression recapitulates fundamental anatomical divisions in the hypothalamus. Neuropeptides preferentially colocalize with metabotropic neurotransmitters, suggesting a system-wide correspondence between slow-acting molecular signaling mechanisms. To investigate the behavioral consequences of distributed neuropeptide systems, we apply meta-analytical decoding to neuropeptide maps and show a spectrum of functions, from sensory-cognitive to reward and bodily functions. Finally, using evolutionary analysis we find extended positive selection for neuropeptides in early mammals, suggesting that refinement of neuropeptides coincides with the emergence of neocortex and higher cognitive function. Collectively, these results show that neuropeptide receptors are highly organized across the human brain and closely intertwined with multiple features of brain structure and function.

## Abstract (web summary)

This study comprehensively maps neuropeptide systems in the human brain to elucidate their organizational principles and shows how neuropeptide architecture is linked to behavior and evolution.

## Main

Neuropeptides are a fundamental molecular signaling system both in the nervous system and the periphery. These neuronal messengers are thought to be mainly synthesized and secreted in a small set of structures, such as the hypothalamus, although there is increasing evidence that both neuropeptides and their receptors are considerably more widespread throughout the brain and body^1,2^. Compared to neurotransmitters, neuropeptides are larger amino acid chains, making them slower to diffuse in extracellular space^3^. Once released, neuropeptides undergo slow enzymatic breakdown, unlike the fast reabsorption associated with classical neurotransmitters^3–5^. However, like metabotropic neurotransmitters, neuropeptides support intercellular communication via G-protein-coupled receptors^3^.

G-protein-coupled receptor signaling is slower than synaptic transmission with transmitter-gated ion channels, and its effects are more sustained^6^. This complexity allows for single molecules to modulate many other downstream channels, such that local signals can be amplified over local circuits and whole-brain networks^5,7^. Consequently, neuropeptide receptor signaling can cascade across neurons and neural populations, enabling widespread, long-lasting changes in circuits up to whole-brain systems. In line with this broad and protracted signaling, neuropeptides support a rich repertoire of functions, including sleep (for example, hypocretin/orexin)^8^, pain (for example, nociceptin/orphanin)^9,10^, feeding (for example, leptin)^11^, reward (for example, opioids)^9,12^ and social cognition (for example, oxytocin)^13–15^. Thus, neuropeptides mediate how the brain perceives and manipulates internal bodily and external environments, coordinating both immediate and enduring neural and behavioral responses.

Despite their importance, how neuropeptide signaling maps onto the organization of the brain remains poorly understood. At the microscale, neuropeptides modulate local neural activity, but how do these microscale changes cascade into mesoscale and macroscale features of brain structure and function? There is a rich literature on the structural and functional properties of individual peptides^8,11,12^, often in circumscribed brain regions, but less is known about the whole-brain organization of this chemical signaling system and how it dovetails with other related systems.

Here we comprehensively mapped neuropeptide systems in the human brain. Using gene transcription as a proxy, we charted the spatial distribution of 38 neuropeptide receptors across 14 different neuropeptide families in both the cortex and subcortex. We then studied how neuropeptide receptors relate to other biological systems in the brain, from molecular and cellular features, to their fine-grained embedding in the hypothalamus, to whole-brain functional specialization. At the microscale, we studied how neuropeptide systems colocalize with classical neurotransmitter systems. At the mesoscale, we associated neuropeptide receptor gene expression with hypothalamic nuclear organization. At the macroscale, we mapped neuropeptide systems onto patterns of whole-brain functional specialization and traced their emergence across phylogeny. Altogether, we demonstrated that the spatial patterning of neuropeptide receptors is highly organized across the brain and has multiple functional consequences.

## Results

**Fig. 1:**
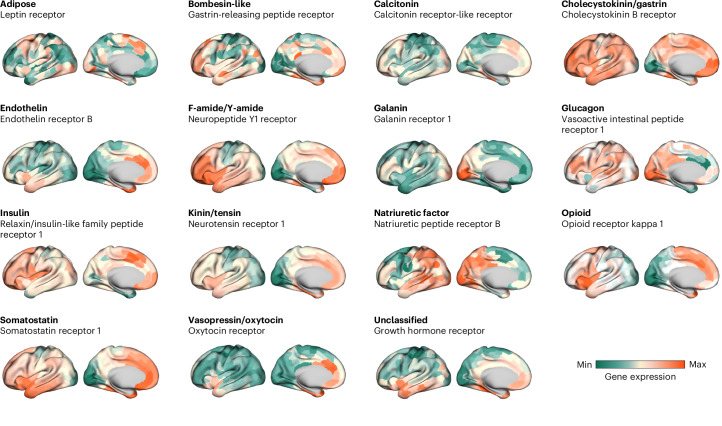
Neuropeptide families. We mapped 38 neuropeptide receptors based on their gene expression available in the AHBA^16^. Of 38 neuropeptide receptors, we identified 14 families of neuropeptides from an established taxonomy of peptides^91^. For each neuropeptide family (bold font), a single exemplar receptor is shown (regular font). Maps are min–max scaled. For simplicity, only cortical topographies are shown, but note that all analyses reported were carried out for both cortex and subcortex. Technical details about gene expression quality control metrics and mean expression in specific structures are shown in Table 1.

A whole-brain atlas of neuropeptide receptors was assembled for 38 neuropeptide receptor-encoding genes across 14 different neuropeptide families (Fig. 1). Topographical receptor maps were reconstructed using the intensity of the corresponding microarray gene transcript from the Allen Human Brain Atlas (AHBA)^16^. Receptor-encoding genes were mapped to the Schaefer 400 cortical atlas^17^, the Melbourne Subcortex Atlas S4 (ref. ^18^) and the hypothalamic delineation from the CIT168 atlas^19^, yielding a whole-brain atlas with 455 regions. Briefly, an initial set of 84 receptor-encoding genes^1^ were filtered based on multiple quality control criteria, including mean intensity, correlation with RNA sequencing (RNA-seq) and differential stability, yielding 38 genes of interest (Table 1 and Methods). To assess the specificity of findings, we compared results with null distributions generated using randomly selected genes with matched spatial autocorrelation and expression magnitude (Methods). We replicated the results with RNA-seq data from the AHBA (Fig. S1) and we systematically compared them with an independent RNA-seq atlas (Human Protein Atlas; Supplementary Fig. 2)^20,21^.

**Table 1:** Neuropeptide receptor overview

Gene	Full name	Family	Quality control			Mean gene expression		
			Mean intensity	Correlation to RNA-seq	Differential stability	Cortex	Hypothalamus	Subcortex
*ADCYAP1R1*	ADCYAP receptor type I	Glucagon/secretin	0.97	0.45	0.35	0.35	0.81	0.64
*ADIPOR2*	Adiponectin receptor 2	Adipose	0.95	0.54	0.14	0.52	0.42	0.60
*APLNR*	Apelin receptor	Unclassified	0.75	0.62	0.30	0.49	0.75	0.56
*BDKRB2*	Bradykinin receptor B2	Kinin/tensin	0.39	0.66	0.40	0.45	0.11	0.32
*CALCRL*	Calcitonin receptor-like receptor	Calcitonin	0.79	0.39	0.18	0.47	0.46	0.44
*CCKBR*	Cholecystokinin B receptor	CCK/gastrin	0.61	0.66	0.54	0.87	0.16	0.28
*EDNRA*	Endothelin receptor type A	Endothelin	0.42	0.60	0.32	0.53	0.32	0.41
*EDNRB*	Endothelin receptor type B	Endothelin	0.96	0.78	0.54	0.40	0.86	0.81
*GALR1*	Galanin receptor 1	Galanin	0.37	0.48	0.38	0.53	0.68	0.37
*GHR*	Growth hormone receptor	Unclassified	0.63	0.70	0.44	0.42	0.69	0.54
*GIPR*	Gastric inhibitory polypeptide receptor	Glucagon/secretin	0.97	0.20	0.15	0.39	0.51	0.56
*GLP2R*	Glucagon-like peptide 2 receptor	Glucagon/secretin	0.31	0.73	0.59	0.67	0.28	0.26
*GRPR*	Gastrin-releasing peptide receptor	Bombesin-like	0.28	0.25	0.27	0.54	0.45	0.21
*HCRTR1*	Hypocretin receptor 1	Unclassified	0.38	0.58	0.18	0.48	0.65	0.50
*LEPR*	Leptin receptor	Adipose	0.76	0.20	0.26	0.37	0.72	0.64
*MCHR1*	Melanin-concentrating hormone receptor 1	Unclassified	0.34	0.58	0.35	0.60	0.19	0.17
*MCHR2*	Melanin-concentrating hormone receptor 2	Unclassified	0.45	0.75	0.42	0.60	0.09	0.10
*NPFFR2*	Neuropeptide FF receptor 2	F-amide/Y-amide	0.51	0.42	0.36	0.49	0.49	0.18
*NPR2*	Natriuretic peptide receptor 2	Natriuretic factor	0.99	0.23	0.19	0.45	0.70	0.51
*NPR3*	Natriuretic peptide receptor 3	Natriuretic factor	0.25	0.42	0.34	0.50	0.53	0.31
*NPY1R*	Neuropeptide Y receptor Y1	F-amide/Y-amide	0.69	0.80	0.66	0.60	0.55	0.45
*NPY2R*	Neuropeptide Y receptor Y2	F-amide/Y-amide	0.29	0.60	0.16	0.47	0.42	0.21
*NPY5R*	Neuropeptide Y receptor Y5	F-amide/Y-amide	0.53	0.45	0.41	0.56	0.54	0.60
*NTSR1*	Neurotensin receptor 1	Kinin/tensin	0.22	0.81	0.67	0.46	0.48	0.31
*NTSR2*	Neurotensin receptor 2	Kinin/tensin	0.98	0.83	0.44	0.44	0.89	0.73
*OPRK1*	Opioid receptor kappa 1	Opioid	0.43	0.71	0.54	0.48	0.53	0.59
*OPRL1*	Opioid-related nociceptin receptor 1	Opioid	0.90	0.65	0.15	0.65	0.57	0.44
*OPRM1*	Mu-opioid receptor 1	Opioid	0.60	0.55	0.62	0.38	0.66	0.58
*OXTR*	Oxytocin receptor	Vasopressin/oxytocin	0.68	0.61	0.44	0.38	0.93	0.66
*RAMP1*	Receptor activity-modifying protein 1	Calcitonin	1.00	0.50	0.21	0.46	0.70	0.72
*RAMP3*	Receptor activity-modifying protein 3	Calcitonin	0.72	0.68	0.51	0.50	0.56	0.36
*RXFP1*	Relaxin/insulin-like family peptide receptor 1	Insulin	0.52	0.67	0.51	0.74	0.12	0.17
*SORT1*	Sortilin 1	Kinin/tensin	1.00	0.46	0.34	0.55	0.16	0.43
*SSTR1*	Somatostatin receptor 1	Somatostatin	0.50	0.86	0.75	0.59	0.66	0.28
*SSTR2*	Somatostatin receptor 2	Somatostatin	0.98	0.80	0.49	0.66	0.31	0.35
*TACR2*	Tachykinin receptor 2	Kinin/tensin	0.71	0.29	0.18	0.49	0.39	0.37
*VIPR1*	Vasoactive intestinal peptide receptor 1	Glucagon/secretin	0.40	0.47	0.35	0.82	0.15	0.32
*VIPR2*	Vasoactive intestinal peptide receptor 2	Glucagon/secretin	0.51	0.85	0.58	0.67	0.29	0.27

Neuropeptide receptor distributions were estimated using gene expression from the AHBA^16^. Gene transcripts were filtered based on multiple quality control criteria, including (1) mean intensity greater than 0.2, (2) correlation with RNA-seq greater than 0.2 and (3) differential stability greater than 0.1. For reference, mean expression within the cortex, hypothalamus and subcortex are shown.

### Mapping neuropeptide signaling in the human brain

Figure 1 shows the cortical spatial distribution for one exemplar receptor in each neuropeptide family. The spatial distributions display considerable heterogeneity and concordance with prior literature. For example, oxytocin receptors are highly expressed in the limbic cortex, which is consistent with their role in social cognition^13,15^. Importantly, there is substantial diversity of receptor expression, motivating a further exploration of how neuropeptide receptors are embedded within the structural and functional organization of the brain.

**Fig. 2:**
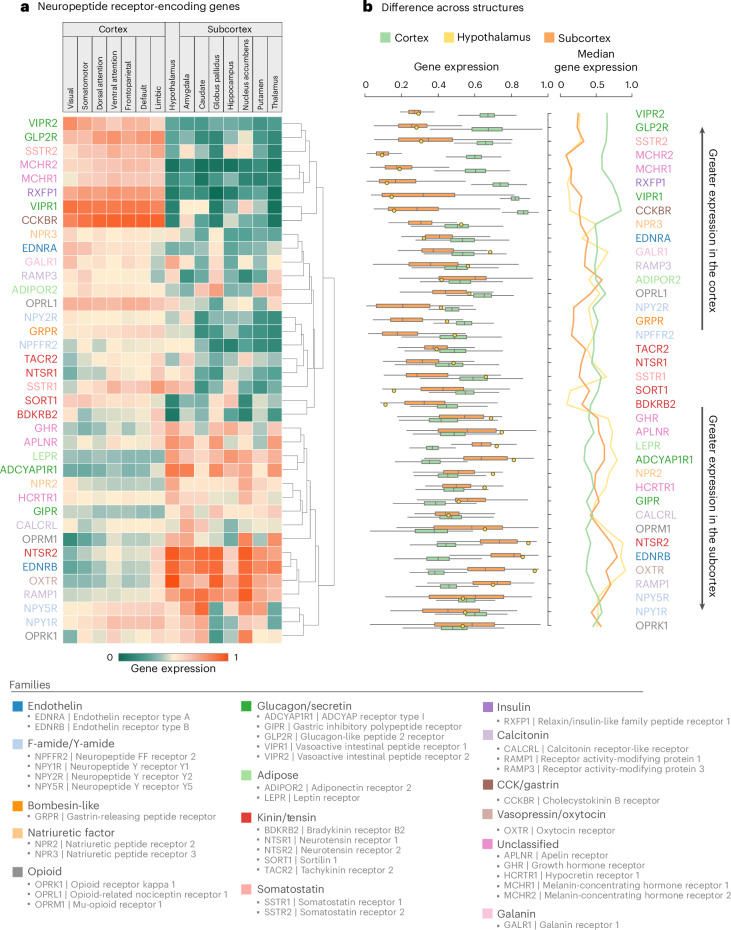
Mapping neuropeptide receptor topographies across the human brain. Neuropeptide receptor topographies stratified according to anatomical and functional landmarks. **a**, Mean expression of individual neuropeptide receptors in the cortex, hypothalamus and subcortex. Cortical receptor expression was stratified according to canonical intrinsic functional networks (visual, somatomotor, dorsal attention, ventral attention, frontoparietal, default mode and limbic)^17^. Subcortical receptor expression was stratified according to a functional atlas (amygdala, caudate nucleus, globus pallidus, hippocampus, nucleus accumbens, putamen and thalamus)^18^. Hypothalamic expression was aggregated from eight nuclei localized in the hypothalamus delineation of the CIT168 atlas^19^. **b**, Box plots of neuropeptide receptor expression within the cortex (*n* = 400), subcortex (*n* = 54) and hypothalamus (*n* = 1), with medians plotted on the right. The bounds of the box plots represent the first (25%) and third (75%) quartiles; the center line represents the median; the whiskers represent the minima and maxima. Receptors are colored according to family membership and vertically sorted using hierarchical clustering.

To more comprehensively describe the anatomical distribution of neuropeptide receptors, we estimated the mean expression of receptor genes in the cortex (stratified according to intrinsic networks), hypothalamus and subcortex (Fig. 2a). The receptors are organized in a cortical–subcortical axis, with some receptors preferentially expressed in the cortex and others expressed in the hypothalamus and other subcortical structures. For example, we observed high expression of oxytocin receptors in the amygdala, opioid receptors in the nucleus accumbens and the leptin receptor in the hypothalamus and hippocampus. Figure 2b summarizes this gradient of expression, showing the median expression of each neuropeptide receptor within the cortex, hypothalamus and subcortex. Interestingly, there are many instances of neuropeptide receptors primarily associated with basal bodily functions but that are nevertheless highly expressed in the brain and in cortex in particular (for example, the cholecystokinin B receptor). Much like vasoactive intestinal peptide (VIP) receptors, which are now known to be expressed in inhibitory cortical interneurons^22^, these examples contribute to a growing realization that, despite their names and initially ascribed functions, neuropeptide receptors can be expressed in the brain and may potentially have additional functions in neural communication^2^. Note that several commonly studied neuropeptide receptors did not pass the quality control criteria and were therefore not considered, such as the main ghrelin receptor (growth hormone secretagogue receptor).

### Neuropeptide axes in hypothalamic nuclei

**Fig. 3:**
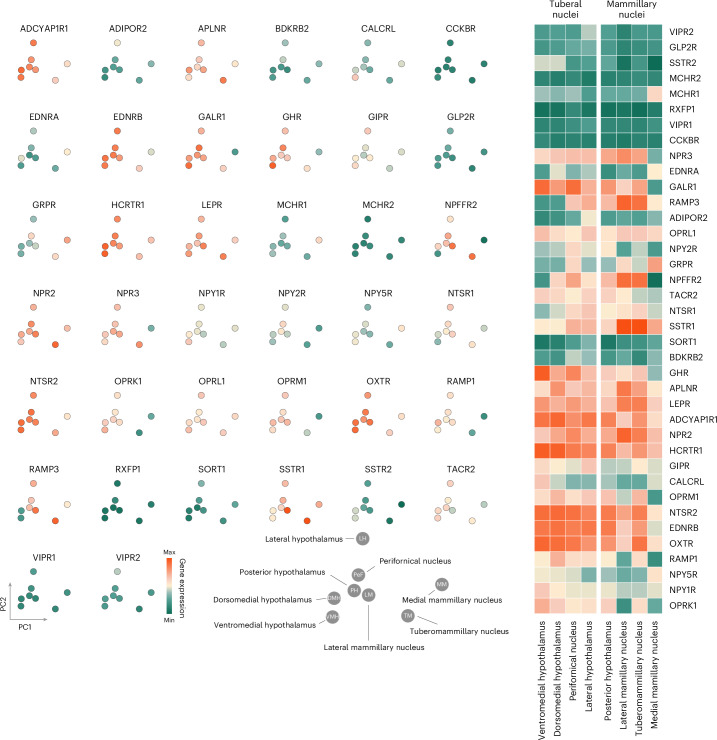
Neuropeptide receptor organization in hypothalamic nuclei. Expression of neuropeptide receptor genes in eight hypothalamic nuclei shown in two-dimensional PCA space. Nuclei include the lateral hypothalamus, the ventromedial nucleus, the dorsomedial nucleus, the perifornical nucleus, the posterior hypothalamus and the mammillary body. The heatmap shows the expression data ordered according to the cluster order in Fig. 2.

We now focused more closely on how neuropeptide receptor organization aligns with the neuroanatomy of the hypothalamus, the nexus of neuropeptide communication^23^. We sampled neuropeptide receptor genes in eight hypothalamic nuclei: the lateral hypothalamus, the ventromedial nucleus, the dorsomedial nucleus, the perifornical nucleus, the posterior hypothalamus, and the medial, lateral and tuberomammillary nucleus (Fig. 3). Given that nuclear divisions of the hypothalamus follow different developmental trajectories and support distinct functions^24^, we asked whether neuropeptide receptor genes are differentially expressed across hypothalamic nuclei.

Principal component analysis (PCA) of receptor gene expression in hypothalamic nuclei revealed two dominant axes of variance in receptor expression. The first principal component (PC1) (45.77% variance explained) captures an anterior–posterior gradient from the tuberal region to the mammillary region (Fig. 3a, abscissae). Nuclei of the tuberal hypothalamus (for example, ventromedial, dorsomedial hypothalamus) loaded opposite to those of the mammillary complex of the hypothalamus, reflecting the anterior–posterior organization of the hypothalamus. This tuberal to mammillary gradient corresponds to distinct developmental programs (trajectories): tuberal nuclei differentiate earlier in embryogenesis, whereas the mammillary body forms later^24^. The second principal component (PC2) (34.87% variance) separates the medial (periventricular) and lateral hypothalamic territories (Fig. 3a, ordinates), mirroring the mediolateral delineation defined by the fornix boundary^25^. PC2 loadings differentiate nuclei within the tuberal region from periventricular midline nuclei (dorsomedial/ventromedial hypothalamus) from the lateral areas of the hypothalamus, consistent with their cytoarchitectural differences in cell and myelin fiber density^25^, which may also stem from different developmental trajectories^26,27^. Together, neuropeptide receptor expression recapitulates fundamental neuroanatomical divisions of the hypothalamus.

### Colocalization with neurotransmitter receptors

**Fig. 4:**
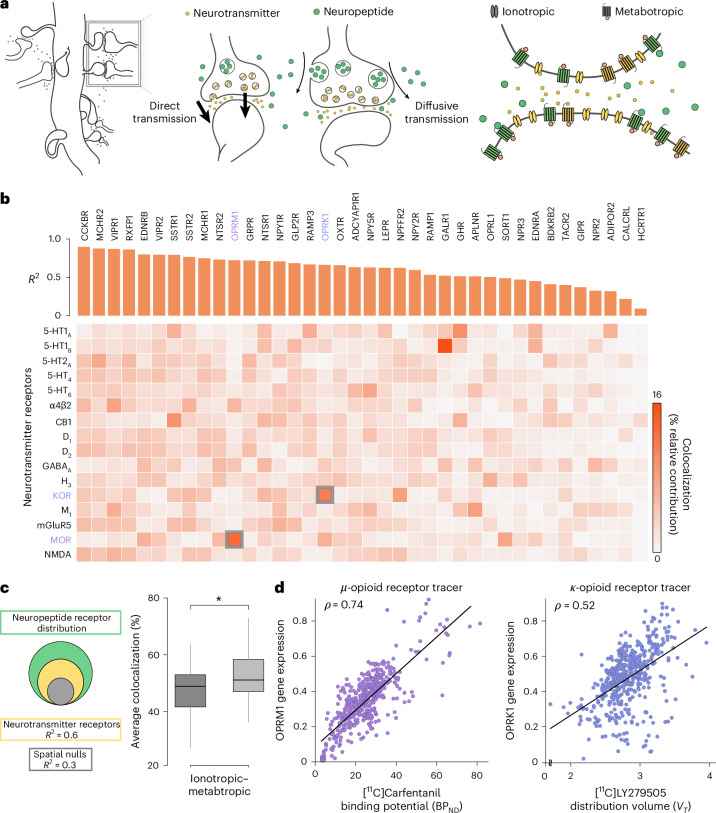
Colocalization with neurotransmitter receptors. **a**, Schematic showing the different types of synaptic chemical signaling, including direct ionotropic and metabotropic neurotransmission and diffusive neuropeptide signaling sharing the same signaling space. **b**, Neuropeptide receptor distributions are correlated (overlap) with 16 neurotransmitter receptor densities estimated using PET imaging^30^. The vertical bars indicate the adjusted *R*^2^ when predicting each neuropeptide receptor map using a linear combination of neurotransmitter receptor maps. Heatmap color intensity corresponds to the relative contribution of each neurotransmitter receptor map to the spatial distribution of a neuropeptide receptor^31^. Note that the color-highlighted receptor-encoding genes have a corresponding PET-estimated map (*μ*-opioid receptor, purple; *κ*-opioid receptor, light blue). **c**, Left: Overall spatial overlap (*R*^2^) between neuropeptide and neurotransmitter receptors. Right: Average colocalization (% relative contribution) of neuropeptide receptors (*n* = 38) to ionotropic and metabotropic neurotransmitter classes. The box plot bounds represent the first (25%) and third (75%) quartiles; the center line represents the median; the whiskers represent the minima and maxima. The single asterisk denotes statistical significance under a two-tailed *t*-test (*t*_(74)_ = 2.76, *P* = 0.007). **d**, Scatter plots showing the spatial correspondence between gene-expression-estimated and PET-imaging-estimated receptor densities of the *μ*-opioid and *κ*-opioid receptors (*μ*-opioid receptor: *ρ*_(452)_ = 0.74, *P*_SMASH_ = 0.0006; *κ*-opioid receptor: *ρ*_(452)_ = 0.52, *P*_SMASH_ = 0.0015). *μ*- opioid and *κ*-opioid receptors were imaged using the [^11^C]Carfentanil and [^11^C]LY2795050 radiotracers, respectively.

Just like neurotransmitters, neuropeptides are chemical messengers that work by binding to G-protein-coupled (metabotropic) receptors, modulating the excitability of proximal cells. The primary difference is that neurotransmitter release is more directed toward the postsynaptic terminal, whereas neuropeptides are secreted more tangentially and diffusely, also binding to receptors located on neighboring cells (Fig. 4a)^5^. Therefore, neurotransmitters and neuropeptides occupy the same local environment, naturally raising the question of how these two systems converge spatially. Given that cotransmission of neurotransmitters and neuropeptides is a fundamental and widespread feature of neuronal communication^28,29^, we investigated how cotransmission at individual synapses translates to the whole-brain level by quantifying the spatial overlap between neuropeptide and neurotransmitter receptors.

In this study, we compared the spatial patterns of all 38 neuropeptide receptors with the spatial patterns of 16 neurotransmitter receptors derived from a whole-brain positron emission tomography (PET) atlas^30^. Notably, the atlas contains both slow-acting metabotropic receptors (for example, 5HT_2a_, D_2_, CB1) and fast-acting ionotropic receptors (α4β2, M1, NMDA, GABA_A_). Figure 4b shows how well the spatial distribution of each neuropeptide receptor can be predicted as a linear combination of neurotransmitter receptor densities. Neuropeptide receptors are ordered according to their adjusted *R*^2^ (orange bars); the heatmap shows the percentage contribution (that is, colocalization) of each neurotransmitter receptor^31^ (Methods). On average, neurotransmitter receptor distributions share 60% spatial variance with neuropeptide receptors (Fig. 4c). When stratified into ionotropic and metabotropic receptors, we found that metabotropic receptors were significantly more colocalized with neuropeptide receptors (*t*_(74)_ = 2.76, *P* = 0.007; Fig. 4c), suggesting that neuropeptide receptors are prevalent in the same synaptic environment where metabotropic neurotransmitter receptors reside.

The availability of PET tracers for two of the 38 receptors studied also offered an opportunity to investigate the extent to which gene expression can be used to quantify receptor density. We focused on *OPRK1* and *OPRM1* genes (corresponding to *κ*-opioid and *μ*-opioid receptors, respectively), for which PET maps are available in the atlas (KOR and MOR; see the color highlights in Fig. 4b). We then examined the spatial correspondence between gene transcription and PET-derived protein densities (Fig. 4d) ^32,33^. We found that spatial correlations between gene transcription and PET-estimated protein densities were both positive and statistically significant, even after accounting for spatial autocorrelation (KOR: *ρ*_(452)_ = 0.52, *P*_SMASH_ = 0.0015; MOR: *ρ*_(452)_ = 0.74, *P*_SMASH_ = 0.0006). Note again that this is a limited comparison, including only two genes of the total 38 in the gene library (‘Discussion’).

### Neuropeptides delineate cognitive domains

In the previous sections, we explored principles that govern the spatial distributions of neuropeptide receptor genes on a microscale and a mesoscale. We next investigated how neuropeptides relate to cognitive specialization on a whole-brain basis. That is, initial discoveries about neuropeptide functions were based on behavioral changes in response to pharmacological manipulation, such as sleep and hypocretin^8^, or weight regulation and leptin^34^. We sought to map regional neuropeptide receptor distributions to patterns of regional cognitive specialization using Neurosynth^35^. Briefly, Neurosynth is a meta-analytical tool that identifies regional activations associated with specific cognitive functions and psychological processes by aggregating data from thousands of functional magnetic resonance imaging (MRI) studies. We cross-referenced Neurosynth terms with terms from the Cognitive Atlas to derive 125 unique meta-analytical maps^36^. The resulting maps represent the likelihood that specific brain regions are associated with a given term, for example, ‘reading’ or ‘eating’, thereby serving as reverse-inference mapping from terms to neural activity.

**Fig. 5:**
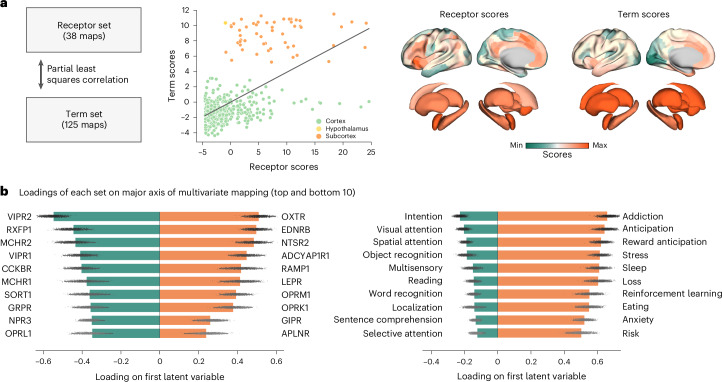
Neuropeptide receptor alignment with cognitive domains. **a**, Partial least squares correlation was used to map the 38 neuropeptide receptor maps to a set of 125 cognitive terms from the Neurosynth meta-analytical atlas^35^. The analysis identified one significant latent variable (*P* = 0.0001). Term and receptor scores are shown as both a scatter plot and surface renderings. **b**, Receptor and term loadings for the first latent variable. The bar plots represent empirical loadings. The error bars represent bootstrap-estimated 95% confidence intervals from *n* = 10,000 bootstrap permutations.

To derive a data-driven mapping between receptors and terms, we used an unsupervised multivariate pattern learning technique, that is, partial least squares correlation (PLSC)^37^. Briefly, PLSC seeks to identify weighted linear combinations of receptors and terms that optimally covary with each other across the brain^38^. We identified a single dominant mapping between receptors and terms (81.17% covariance accounted for, mean out-of-sample correlation *r*_(453)_ = 0.36; Fig. 5a), and test it against (1) 10,000 spatial-autocorrelation-preserving null sets (*P* = 0.0001)^39^ and (2) 10,000 sets of random genes with matching spatial autocorrelation and value distribution (Methods; *P* = 0.0001). This common axis displays a division between cortex and subcortex as evidenced by the disjoint distributions of scores, and is reminiscent of the cortex–subcortex gradient of neuropeptide receptors observed earlier (Fig. 2). In other words, cognitive architecture mirrors the underlying chemoarchitectural gradient.

To understand the specific receptor–term mappings that constitute this gradient, we inspected the receptor and term loadings, that is, the degree to which receptors and terms are correlated with the latent variable (Fig. 5b). On the negative side, cortically expressed neuropeptide receptors like VIPR1 and VIPR2, MCHR1 and MCHR2, RXFP1 and CCKBR (negative bars on the left) covary with sensory processing terms such as ‘visual/spatial attention’, ‘object recognition’, ‘reading’ or ‘localization’. On the positive side, more subcortically expressed receptors (OXTR, EDNRB, NTSR2, ADCYAP1R1, RAMP1, LEPR, OPRM1) covary with terms such as ‘addiction’, ‘anticipation’ and ‘risk’. These receptors and terms are prominent in the reward and reinforcement learning literature^9,40–43^, which involves the mesolimbic (predominantly subcortical) reward circuit^42,44,45^. Additionally, positive loadings for terms related to emotional regulation such as ‘stress’, ‘loss’ or ‘anxiety’, and basal bodily functions such as ‘eating’ or ‘sleep’. Collectively, these results show that the anatomical layout of neuropeptides in the brain is closely associated with a spectrum of basal bodily to higher cognitive functions. This differentiated function of neuropeptides may potentially reflect homeostatic and allostatic demands rooted in environmental demands and evolutionary adaptation^46^, a question that we pursue in the next subsection.

### Evolution of molecular signaling systems

**Fig. 6:**
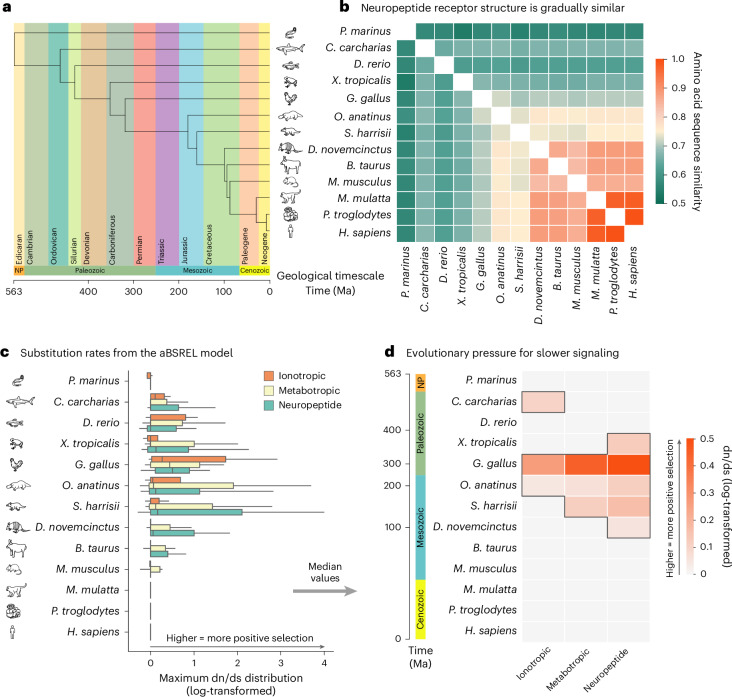
Neuropeptide signaling across phylogeny. **a**, Phylogenetic lineage of human evolution for 13 species, each representative of a specific era in human evolution (for example, first vertebrate, first tetrapod)^14,92^. **b**, Amino acid sequence (AAS) similarity of neuropeptide receptors across evolution. **c**, Codon-based substitution rates for receptor genes, stratified according to ionotropic (*n* = 13) and metabotropic (*n* = 23) neurotransmitters, and neuropeptides (*n* = 33). The box plot bounds represent the first (25%) and third (75%) quartiles; the center line represents the median; the whiskers represent the minima and maxima. **d**, Median substitution rates for different types of neuropeptide and neurotransmitter receptors. All values are log-transformed. Supplementary Table 1 shows the exact statistical estimated and fitted parameters of the aBSREL model. **a**,**c**, Icons were reproduced from The Noun Project (CC BY 3.0). Credits: L. Meiertoberens (*P. marinus*), Hey Rabbit (*C. carcharias*, *D. novemcinctus* and *P. troglodytes*), L. Lortal (*D. rerio*), Brand Mania (*X. tropicalis*), J. K. Lim (*G. gallus*), Parkjisun (*O. anatinus*), Vanicon Studio (*S. harrisii*), Hat-Tech (*B. taurus*), Econceptive (*M. musculus* and *M. mulatta*), Sabil (*H. sapiens*).

To investigate the evolutionary roots of neuropeptide signaling, we concluded by conducting an exploratory evolutionary analysis focusing on a set of 12 species separated from *Homo sapiens* at distant points in the past. These species were selected to represent key points along the vertebrate lineage^14,15^, which are: *Petromyzon marinus* (sea lamprey), *Carcharodon carcharias* (great white shark), *Danio rerio* (zebrafish), *Xenopus tropicalis* (western clawed frog), *Gallus gallus* (chicken), *Ornithorhynchus anatinus* (platypus), *Sarcophilus harrisii* (Tasmanian devil), *Dasypus novemcinctus* (nine-banded armadillo), *Bos taurus* (cattle), *Mus musculus* (house mouse), *Macaca mulatta* (rhesus macaque) and *Pan troglodytes* (chimpanzee) (Fig. 6a). Species relatedness among vertebrates is reflected in the gradually increasing amino acid similarity of neuropeptide receptors (Fig. 6b). However, while molecular similarity identifies shared ancestry^47^, it does not identify the specific points in evolution at which adaptive changes occurred^48^.

We investigated the evolutionary moments when neuropeptide receptors were preferentially selected during evolution, that is, events of positive selection throughout the human lineage. To contextualize neuropeptide signaling evolution, we additionally investigated convergences of other signaling receptors, namely ionotropic and metabotropic neurotransmitter receptors (see Methods for a list of genes). We applied an adaptive branch-site random effects likelihood (aBSREL) model to parse the rate of nonsynonymous versus synonymous changes (substitution rate dn/ds) in the genome as a measure of positive selection (Methods)^49^. In other words, the aBSREL model allowed us to infer stages in evolution associated with positive selection of receptor genes. Figure 6b shows the distribution of log-transformed substitution rates for ionotropic and metabotropic neurotransmitter, and the neuropeptide receptor categories for our ancestor species. In this context, substitution rates above zero indicate positive selection, values at zero indicate neutral evolution and negative values indicate purifying selection. Figure 6c shows the distribution of substitution rates among signaling types. The genetic evolution of signaling genes is characterized by periods of intense selection followed by relaxation at different rates for each signaling type. Specifically, there is a significant effect of signaling type on substitution rates (Friedman *χ*^2^_(2)_ = 7.54, *P* = 0.023, Kendall’s *W* = 0.29), indicating moderate variability in positive selection between ionotropic, metabotropic and neuropeptide genes. Inspecting the distribution medians, we observed that receptor-encoding genes collectively exhibited a maximum in positive selection around *G. gallus* and *O. anatinus* (Fig. 6d). Once again, we observed that each signaling type differed in their evolutionary trajectory, with positive selection for ionotropic genes slowing down before metabotropic neurotransmitter and neuropeptide genes. Taken together, this suggests that signaling genes around the transition from amniotes (*G. gallus*) to mammals (*O. anatinus*) may have conferred an evolutionary advantage, with sustained selection for slower modulatory signaling during early mammalian evolution. Interestingly, compared to neurotransmitter receptors, we found that peptide receptors undergo positive selection across multiple contiguous stages of evolution. This evolutionary differentiation of genes is also mirrored by their anatomical localization: when inspecting neuropeptide genes that are positively selected, we observed that genes selected in earlier evolutionary branches are now predominantly found in the subcortex, for example, *APLNR*, *EDNRB* and *NPR2*. In contrast, neuropeptide genes that show selective pressure in more recent stages of evolution, such as *MCHR1*, *VIPR2*, *NPFFR2* and *GRPR*, are expressed foremost in the cortex. Altogether, these results suggests that the cortex–subcortex differentiation observed in the previous sections may have an evolutionary origin and underscore the concomitant emergence of neuropeptide signaling, mammalian cortical development and higher cognitive function.

### Sensitivity and specificity

Finally, we sought to evaluate the sensitivity and specificity of the results across additional datasets and alternative methodological choices. First, we verified the correspondence between gene expression and two available PET measurements of protein abundance (Fig. 4). Second, we replicated the main results using RNA-seq data, both from AHBA (Supplementary Fig. 1) and from the independently collected Human Protein Atlas (Supplementary Fig. 2). Third, we repeated the analyses using an alternative parcellation based on anatomical landmarks^50^, finding an equivalent gene expression distribution (Supplementary Fig. 3). Fourth, the results were contrasted with multiple null models, including spatial-autocorrelation-preserving randomization and matched gene nulls (Methods).

In addition to these sensitivity analyses, we also conducted three sanity checks based on domain knowledge about neuropeptides. First, we found that neuropeptides previously known to display sexual dimorphism, such as relaxin^51^, melanin-concentrating hormone^52^ and oxytocin^53^, also have differential expression between male and female donors (Supplementary Fig. 4), which is consistent with their role in reproduction^54^. Second, we found substantial spatial correlations between endothelin receptors, known to regulate blood supply to stay within healthy physiological ranges^55,56^, and regional blood flow estimated using arterial spin labeling (ASL) (Supplementary Fig. 5). Third, using only Neurosynth maps related to ‘feeding’ and ‘eating’, we found notable enrichment for neuropeptides known to modulate appetitive behavior (Supplementary Fig. 6d), such as leptin^57,58^ and calcitonin^59,60^.

## Discussion

Neuropeptides constitute a fundamental chemical communication mechanism throughout the nervous system and the entire body^4,61^. These signaling molecules are released under sustained neural activity and remain diffused through extracellular space for longer periods^3,23,61^. Their slow, global presence in circuits has drawn increasing attention to them as key regulators of states beyond local synaptic signaling^4,23,62^. Despite their importance, neuropeptide signaling remains uncharted territory, with only recent efforts trying to map its presence at the systems level^2,63^. In this study, we comprehensively mapped whole-brain human neuropeptide signaling across multiple levels of description, from molecular and cellular embedding to mesoscale nucleus organization in the hypothalamus and macroscale whole-brain cognitive specialization. As we elaborate in this section, neuropeptide signaling is highly organized and leaves an indelible mark on several features of brain structure and function.

Spatially, neuropeptide systems exhibit a striking anatomical organization across cortical and subcortical regions. For instance, receptors for somatostatin, VIP and melanin-concentrating hormone are predominantly expressed in the cortex, while other families, such as neuropeptide Y (NPY), opioid, natriuretic and endothelin receptors, are expressed across both cortical and subcortical regions. In contrast, receptors for neuropeptides like oxytocin and calcitonin are more restrained to subcortical nuclei. This anatomical gradient suggests a potential specialization in the roles had by different neuropeptides, with some potentially modulating cortical and subcortical circuits together, while others act more focally within one structure.

Our findings in the hypothalamus illustrate how neuropeptide receptor architecture is specified by development and subsequently used for diverging functions. We show that neuropeptide receptor genes vary along two principal axes, a tuberomammillary axis and a mediolateral axis, mirroring the developmental blueprints that later shape anatomical divisions in the hypothalamus^27,64^. These molecular axes map onto distinct roles: tuberal nuclei coordinate feeding, energy balance and thermoregulation, whereas mammillary complexes engage arousal and memory-related limbic circuits^24,64^. Beyond its internal organization, the hypothalamus sits at the crossroads of neural and hormonal communication, with rich connections to the cortex and subcortex^65,66^, and specialized interfaces to the periphery, such as the median eminence and pituitary stalk^24^. Through these conduits, the hypothalamus uses neuropeptide signaling as a shared chemical language to integrate neural computations with peripheral physiology^64,67^. Thus, neuropeptide receptor organization in the hypothalamus constitutes a scaffold linking developmental trajectories to functional divisions and, ultimately, to brain–body communication^27^. As we outline below, the confluence of gross anatomical localization, signaling environment and prolonged timescale of effect may altogether confer neuropeptides the capacity to modulate a variety of bodily and cognitive functions.

At the molecular level, we show that neuropeptide receptors tend to colocalize with predominantly metabotropic, and, to a lesser extent, ionotropic neurotransmitter systems. This arrangement is consistent with the notion that neuropeptides primarily act as a global modulation architecture to facilitate or restrict local G-protein-coupled plasticity from metabotropic neurotransmitter activity^68,69^. This is akin to previous theoretical frameworks of meta-plasticity and three-factor learning, where a third modulatory factor influences traditional Hebbian learning^68,70,71^. To a lesser degree, neuropeptides may also prime local ionotropic activity, enabling or disabling a milieu of excitable cells to promote spike-timing-dependent plasticity^68^. In this study, previous literature elucidated the computational flexibility that arises from cotransmission of fast (ionotropoic) and slow (neuropeptide) signaling^29,72^, providing a biological grounding of the multiplexed signaling environment observed in the brain. Thus, together, the manifold of communication protocols may endow a repertoire of functions, ranging from fast ionotropic communication to slow, sustained neuropeptide state signaling to allow for both immediate responses and long-term adaptability^68,73,74^.

At the systems level, we show that neuropeptides colocalize with whole-brain patterns of cognitive specialization. Specifically, we found that subcortical neuropeptide receptors are associated with behavioral states related to homeostasis and state regulation, including reward anticipation, reinforcement learning, stress, anxiety, sleep and eating. In contrast, cortical neuropeptide receptors are linked to functions related to cognition, such as visuospatial attention, multisensory integration and intention. This underlines the role of neuropeptides as systemic regulators, and it is one of several recent studies investigating how molecular features shape regional cognitive specialization^30,38,75^. Indeed, many neuropeptides are hormonally released in both the brain and the periphery, serving a double function in regulating both physiology and behavior^4,5^. Thus, the concomitant molecular–anatomical differentiation of function, with cortical peptides supporting sensory-cognitive and subcortical modulating basal bodily processes, may potentially reflect the evolutionary history of the nervous system, a question we consider next.

From an evolutionary perspective, refinement of neuropeptide and metabotropic neurotransmitter signaling increases during the transition from amniotes to early mammals. We found heightened selection for neuropeptide receptor genes during this period, with those positively selected before the emergence of the cortex predominantly localized in the subcortex. With the advent of mammals, later-selected neuropeptide receptors are now found in the cortex, supporting the idea that that neuropeptide signaling location can be in part explained by the timing of its evolutionary pressure. Moreover, we found that the evolutionary pressure for metabotropic and neuropeptide signaling is more protracted around the advent of mammals than for ionotropic, suggesting a continued refinement of their function during this time. Indeed, neurons might have evolved to express neuropeptides because of their ability to produce multiple peptides from one single precursor gene, thereby facilitating signaling diversity while being able to regulate peptide activity through enzymatic modifications that respond to cellular conditions^3,61^. Together with the knowledge that peptides are an ancient signaling mechanism^2,14^, we hypothesize that neuropeptides have been co-opted for a wide range of functions throughout evolution, ranging from basal cellular regulation to higher cognitive computations.

These results are founded on an important methodological assumption: that transcript quantity is correlated with translated protein quantity. The bulk microarray measurements used in this study are limited in resolution and may not perfectly correspond to regional receptor protein density, a mismatch we partially mitigated through stringent probe selection and cross-referencing with in and out-of sample RNA-seq data (Supplementary Figs. 1 and 2). Moreover, any correspondence between mRNA and receptor density is conditioned by multiple regulatory processes, from transcription through translation to eventual receptor availability^76^. These include pretranscriptional and posttranscriptional mechanisms like RNA splicing, translation efficiency, proper protein folding, posttranslational modifications, trafficking to the appropriate membrane compartment and dynamic insertion into the membrane^33,76,77^. Together, these steps determine functional receptor abundance and can decouple mRNA levels from protein density. Such decoupling is further shaped by region-specific and subunit-specific factors like stoichiometry and trafficking constraints, as well as epigenetic regulation and differential transcript half-lives, all of which can weaken gene-to-protein translation across different human brain tissues^76,78,79^. At the same time, there is also evidence that receptor-encoding gene expression can track protein distributions for specific systems and targets^32,77,79,80^. Consistent with this, we observe gene–protein spatial correlations for two (opioid) receptors where PET measurements are available. Whether these spatial correspondences between neuropeptide genes and receptors are universal is currently unknown and an important avenue for future research. Looking ahead, whole-brain, cell-type-resolved measurements will be critical to evaluate gene–protein relationships in neuropeptide systems; in particular, genetically encoded G-protein-coupled receptor activation sensors^81^ and advances in PET radiochemistry^82^ promise more precise in vivo assays of peptide signaling and receptor density that can be integrated with high-quality transcriptomics to resolve these ambiguities.

In addition, the present findings should be interpreted in light of several methodological limitations. First, gene expression estimates vary with demographic factors such as sex, age and environmental influences^83,84^. Although the AHBA remains the most spatially comprehensive transcriptomic resource, it consists of only six donors with uneven sampling of biological sex. While we attempted to study sex differences in neuropeptide receptor expression and found results consistent with the literature, these findings are limited by the available data. Further, neuropeptide abundance varies as a function of individual states of hunger^85^, stress^86^ or arousal^87^. This underscores the need for more extensive and more balanced sampling for making stronger inferences about interindividual and intraindividual variability in receptor expression^88,89^. As more comprehensive datasets become available, particularly those capturing more diverse populations^90^, these factors will open up exciting new avenues for research in the future.

In conclusion, we show that neuropeptides represent an omnipresent component of signaling within the human brain, acting at multiple scales to influence both local synaptic dynamics and global brain states. Their spatial distribution across cortical and subcortical regions reflects their diverse roles from modulating cognition to bodily states. Likewise, the coincident positive selection for neuropeptides during the emergence of the neocortex underscores the putative contribution of this molecular signaling mechanism to the complexity and adaptability of human neural circuits. Thus, neuropeptides, through their long-lasting and widespread effects, can be seen as key players in the coordination of both immediate and enduring neural and behavioral responses.

## Methods

The code to perform all analyses is available at https://github.com/netneurolab/ceballos_neuropeptide-organization, with data available at https://osf.io/4rsz9/.

### Gene expression data

We begin with the neuropeptide receptor atlas used for the main analyses. Our curated atlas is based on gene expression data from the AHBA (*n* = 6, one female, aged 24–57, three of White ethnicity, two African American, one Hispanic^16^) using the abagen toolbox (v.0.1.4, https://github.com/rmarkello/abagen)^93^. Detailed information about data acquisition can be found in ref. ^16^. Informed consent was obtained from each decedent’s next-of-kin.

Preprocessing of gene expression data closely followed the procedures described in ref. ^94^. Microarray probes for all individuals were fetched from https://human.brain-map.org/and re-annotated to match the gene symbol ID and name from the latest version of the NCBI^95^, found under https://ftp.ncbi.nih.gov/gene/DATA/GENE_INFO/Mammalia/. Probes not matched to a valid ID were discarded. The remaining microarray probes were subjected to intensity-based filtering, where probes with intensity less than background noise in less than 20% of samples across individuals were excluded. For genes that had multiple probes assigned, the probe with the highest average Pearson correlation to the RNA-seq data from two individuals in the dataset was selected. This ensured biological plausibility of the microarray measurements wherever possible^96^. Tissue sampling locations from the original T1-weighted scans of individuals were then nonlinearly registered to the Montreal Neurological Institute 152 1-mm space as in https://github.com/chrisgorgo/alleninf. Probe samples were assigned to brain regions in a standard atlas consisting of the seven-network Schaefer 400 atlas^17^ and the Melbourne Subcortex Atlas^18^. As the latter does not include the hypothalamus, we additionally used the hypothalamic delineation from the CIT168 atlas^19^, thresholded at a 0.5 probability. Samples were then assigned to a brain region if their Montreal Neurological Institute coordinates were within a 2-mm vicinity. To reduce the potential of misassignment, sample-to-region matching was constrained by hemisphere and gross structural divisions (that is, cortex, subcortex/brain stem and cerebellum, such that, for example, a sample in the left cortex could only be assigned to an atlas voxel in the ipsilateral side). If a brain region was not assigned a sample based on that procedure, every voxel in the region was mapped to the nearest tissue sample from the individual to generate a dense, interpolated expression map. The mean of these expression values was taken across all voxels in the region, weighted according to the distance between each voxel and the sample mapped to it, to obtain an estimate of the parcellated expression values for the missing region. Interindividual variation was addressed by normalizing sample expression values across genes using a robust sigmoid function as in ref. ^97^. Normalized expression values were then rescaled from 0 to 1 using min–max normalization. Gene expression values were subsequently normalized across regions using an identical procedure. All available tissue samples were used in the normalization process regardless of whether they were assigned to a brain region. Tissue samples not matched to a brain region were discarded after normalization. Samples assigned to the same brain region were averaged separately for each individual, which resulted in six expression matrices, one for each individual, with rows corresponding to brain regions and columns corresponding to genes. All matrices were finally mean-averaged across individuals, resulting in a single matrix representative of the expression level of a particular gene in a given region.

### Receptor PET data

PET-derived receptor density maps were collated by Hansen et al.^30^ and downloaded from neuromaps (v.0.0.4) (https://github.com/netneurolab/neuromaps)^98^ for 16 neurotransmitter receptors and transporters across eight neurotransmitter systems. These include dopamine (D_2_)^99^, serotonin (5-HT_1A_^32^, 5-HT_1B_^100^, 5-HT_2A_^32^, 5-HT_4_^32^, 5-HT_6_^101,102^), acetylcholine (α4β2^103^, M1 (ref. ^104^)), glutamate (mGluR5)^105^, GABA (GABA_A_^79^), histamine (H_3_)^106^, cannabinoid (CB1)^107^ and opioid (MOR^108^, KOR^109^). Methodological details about each tracer can be found in the neuromaps documentation (https://netneurolab.github.io/neuromaps/listofmaps.html) or in the original report of the receptor library^30^. Volumetric PET images were parcellated according to the Schaefer 400 region^17^, Melbourne Subcortex S4 (ref. ^18^) atlas and the hypothalamus delineation from the CIT168 atlas^19^.

### Dominance analysis

To assess colocalization between classical neurotransmitters and neuropeptide receptors, we opted to use dominance analysis^31^, a multilinear regression technique, for two reasons. First, it allowed us to include every available neurotransmitter receptor map simultaneously to predict the spatial expression of neuropeptide receptors. Second, dominance analysis quantifies the prediction performance of a neurotransmitter receptor by building subsets of the full multiple linear regression model and calculating the average contribution of a neurotransmitter receptor when being included into all possible models. This results in a measure of the total (general) dominance^31^ a neurotransmitter receptor has on the spatial expression of a neuropeptide receptors. The sum of all total dominances is equivalent to the *R*^2^ of the full model, which means that the total dominance of each neurotransmitter receptor can be normalized by its sum to obtain the percentage it contributes to the total *R*^2^, also known as relative contribution, which can then be turned into a percentage of the total spatial variance explained by a neurotransmitter receptor. In other words, we are able to answer the question of how much more in line are the distributions of neurotransmitter and neuropeptide receptors when including a particular neurotransmitter receptor into the multilinear model. Finally, to test whether there is a difference in the percentage explained by ionotropic and metabotropic neurotransmitter receptor densities, we first averaged the total dominance of each and then compared the relative contribution of the two classes to the total sum *R*^2^. Dominance analysis was implemented in netneurotools (v.0.2.5)^110^.

### Spatial autocorrelation-preserving null

For all analysis comparing the spatial distribution between brain maps, we used BrainSMASH^39^ as implemented in the neuromaps^98^ library. Briefly, BrainSMASH is a null model to generate surrogate brain maps that match a target spatial autocorrelation^39^. The model entails two steps: (1) random permutation of values within a designated brain map; and (2) smoothing and rescaling to restore the spatial autocorrelation characteristic of the target data. In the first step, values within the brain map are randomly permuted. The permuted data are then transformed to reintroduce spatial autocorrelation. This transformation involves combining the permuted data and a normally distributed random variable. Then, ratios in this combination are optimized using least squares to match the spatial variance patterns (variograms) of the target and surrogate data.

### Random matched gene null set

To assess whether the observed spatial autocorrelation patterns of neuropeptide receptor genes were unique or consistent with random gene expression patterns, we instantiated a random sampling approach to contrast our results to other genes that match the spatial expression of a given neuropeptide receptor based on two criteria: spatial autocorrelation and expression value distribution. This procedure was repeated 10,000 times for each of the 38 neuropeptide receptors that passed our quality control protocol. We outline our approach below.

First, we randomly selected *n* = 100 genes from all genes identified in the AHBA to create a random subset of genes. This set excluded neuropeptide receptor and precursor genes. To ensure a close match in spatial autocorrelation, the Moran’s I of each gene in the subset was computed and compared to the corresponding value of the neuropeptide receptor gene^111^. The Kolmogorov–Smirnov statistic was then estimated to assess how well the expression distribution of the random genes matched that of the neuropeptide receptors. For each neuropeptide receptor gene, we ranked the difference between its Moran’s I and that of each null gene, and the Kolmogorov–Smirnov statistic between their expression distributions. The null gene with the best average rank between the two criteria was then chosen as the closest match and recruited into the null distribution.

### Neurosynth meta-analytical term data

We sought to functionally contextualize our collection of neuropeptide receptors using meta-analytical databases. Maps of functional-behavioral processes were obtained from Neurosynth^35^, a meta-analytical tool that synthesizes results from more than 14,000 published functional MRI studies by searching for high-frequency terms (such as ‘pain’ and ‘attention’) that are published alongside functional MRI voxel coordinates. We modeled activation based on activation likelihood estimation^112^, which estimates the consistency in which a voxel is reported for a given term across studies, highlighting brain regions with increased likelihood of being associated with that term. Although more than 1,000 functional-behavioral processes are reported in Neurosynth, we focused primarily on cognitive function, therefore limiting the terms of interest to cognitive and behavioral terms. These terms were cross-referenced with the Cognitive Atlas, a public ontology of cognitive science^36^, which includes a comprehensive list of neurocognitive processes. We used 125 terms, ranging from umbrella terms (‘attention’ and ‘emotion’) to specific cognitive processes (‘visual attention’ and ‘episodic memory’), behaviors (‘eating’ and ‘sleep’) and emotional states (‘fear’ and ‘anxiety’). The coordinates reported by Neurosynth were parcellated according to the Schaefer 400 region^17^ and Melbourne Subcortex S4 (ref. ^18^) atlases. Additionally, we included the hypothalamus delineation from the CIT168 atlas^19^. The full list of all terms is shown in Supplementary Fig. 6.

### Partial least squares correlation

We applied partial least squares correlation (PLSC) to investigate the relationship between behavioral term maps derived from Neurosynth (*X* matrix) and the gene expression profiles of neuropeptide receptors (*Y* matrix)^37^. Both matrices were centered and scaled to unit variance in each column to ensure comparability. PLSC identifies latent variables that capture the maximum shared variance between two datasets by performing singular value decomposition on the cross-covariance matrix *X*^*T*^*Y*. This process was carried out using a Python implementation of PLSC (behavioral_pls), openly available at https://github.com/netneurolab/pypyls (v.0.0.2).

The decomposition yields singular values that reflect the strength of the covariance explained by each latent variable pair, and singular vectors that weigh the contribution of the original variables (that is, neuropeptide receptor expression and term activations). The latent weights were applied to the respective matrices to obtain the PLSC scores, which represent the data projected onto the latent components. Loadings, representing the contribution of each map to the latent variable, were computed by assessing the similarity of each map to its respective score using Pearson correlation. This allows us to interpret how individual behavioral term maps and gene expression profiles contribute to each latent component.

To assess the significance of the identified latent components, we used two distinct permutation testing strategies. First, we generated a set of random null maps that preserved the spatial autocorrelation properties of the neuropeptide receptor maps and shuffled the *Y* matrix accordingly^38^. Second, we exchanged the *Y* matrix with a set of random matching genes that maintained the key properties of the gene expression data (see ‘Random matched gene null set’). For each permutation test, we computed the singular values of the cross-covariance matrix for the shuffled data, thereby generating null distributions of singular values. The significance of the actual singular values was determined by comparing them to the null distributions derived from both procedures.

To ensure the generalizability of the PLSC results, we performed cross-validation by randomly splitting the observations into training and test sets 1,000 times^113,114^. The training set included 70% of the observations; the PLSC was repeated using this subset. Goodness of fit was evaluated by calculating the correlation between the scores of *X*_training_ and *Y*_training_. The projection weights derived from the training set were then applied to the remaining 30% in the test set, transforming the test observations into scores. We similarly assessed the correlation between the scores of *X*_test_ and *Y*_test_ to evaluate how well the PLSC model generalized to unseen data.

### Evolutionary analysis

To investigate signatures of positive selection in ionotropic, metabotropic and peptide signaling genes, we used a modified procedure based on the original analysis of ref. ^14^. We focused on receptor genes associated with ionotropic (*CHRND*, *GRIA1*, *CHRNA1*, *HTR3A*, *GRIK1*, *CHRNG*, *GABRG1*, *GABRA1*, *CHRNE*, *GRIN1*, *GABRA2*, *GABRG2*, *CHRNB1*) and metabotropic signaling (*GABBR1*, *HRH2*, *DRD2*, *ADRB2*, *CHRM2*, *DRD4*, *HRH4*, *ADRA1A*, *CHRM1*, *ADRB1*, *MTNR1A*, *HTR4*, *HTR2A*, *HRH1*, *DRD1*, *GABBR2*, *GRM1*, *ADRB3*, *CHRM3*, *ADRA2A*, *HTR1A*, *DRD3*, *HRH3*), and compared those to the ensemble of neuropeptide receptor genes previously used to contextualize our results.

Orthologous AASs and coding sequences (CDS) for the selected genes were manually downloaded from the NCBI database for 13 vertebrate species: *H sapiens* (human), *P. troglodytes* (chimpanzee), *M. mulatta* (rhesus macaque), *M. musculus* (house mouse), *B. taurus* (cattle), *D. novemcinctus* (nine-banded armadillo), *S. harrisii* (Tasmanian devil), *O. anatinus* (platypus), *G. gallus* (chicken), *X. tropicalis* (western clawed frog), *D. rerio* (zebrafish), *C. carcharias* (great white shark) and *P. marinus* (sea lamprey). These species represent key evolutionary lineages within vertebrates and provide a comprehensive framework for identifying evolutionary signatures across a broad phylogenetic spectrum^14^.

For each gene, the collected AASs and CDS from the different species were compiled into a single protein FASTA file and a CDS transcript FASTA file. The protein sequences in each file were aligned using MUSCLE (v.5.1)^115^ to create multiple sequence alignments for each receptor gene. These alignments were then translated into codon-based alignments using PAL2NAL (v.14)^116^, which converts protein sequence alignments into nucleotide alignments while preserving codon structure. The resulting codon alignment files were further processed by inserting dummy sequences for missing orthologs, renaming sequence identifiers to abbreviated taxon names (for example, ‘*H. sapiens*’ became ‘*hsapiens*’), and converting the FASTA format codon alignments to PHYLIP sequential format files using TriFusion (v.1.0.1)^117^. We report the similarity of amino acids from the PHYLIP sequential format files as computed in TriFusion.

In addition, a vertebrate species tree was generated using TimeTree^92^, incorporating data from published studies to produce a reliable species tree. The tree was downloaded in Newick format, with branch lengths manually removed to match the format required for subsequent analyses. Taxon names in the tree file were shortened to match those in the codon-based alignments.

Positive selection analyses were conducted using the aBSREL model from the HyPhy package (v.2.5.52)^118,119^. The aBSREL method is an exploratory tool for detecting episodic positive selection across all branches in an evolutionary tree without the need for specifying foreground branches a priori. This model compares a full, alternative model against a null model and performs a likelihood ratio test to identify the magnitude that the empirical results exceed chance (see ref. ^49^ for a detailed description of the aBSREL method). Our main results are based on the categorical median substitution rates, also known as dn/ds or omega ratios, between ionotropic, metabotropic and peptide receptor genes. Substitution rates are informative in this context because they provide insight into the evolutionary pressure exerted on genes, allowing us to differentiate between genes that have undergone positive selection and those that have remained under purifying selection at a particular branch. By comparing these rates across signaling types, we can infer the relative evolutionary dynamics and selective pressures that have shaped neuropeptide functionality.

### Friedman test

To investigate whether the substitution rates (dn/ds) varied significantly across the three signaling categories—ionotropic, metabotropic and peptides—a nonparametric Friedman test^120^ was performed using Pingouin (v.0.5.4)^121^. This test was chosen because of its suitability for repeated measures data, where dn/ds values were modeled for 13 branches representing species in the human lineage. We chose to model the categorical difference between signaling types, treating each branch as a repeated measure across categories, to account for phylogenetic relatedness and shared evolutionary history. This approach enabled a robust assessment of differences in median substitution rates across categories without assuming normality in the data distribution. Significance was determined at *P* < 0.05, and effect size was calculated using Kendall’s *W* to quantify the strength of association between gene category and substitution rate variation^122^.

### Human Protein Atlas RNA-seq data

We used an independently collected dataset from the Human Protein Atlas to cross-check the spatial profile of neuropeptide receptor expression. Human Protein Atlas gene expression data are based on RNA-seq analysis of micropunch samples collected from the Human Brain Tissue Bank at Semmelweis University, Hungary, covering 190 distinct brain regions, areas and subfields^20,21^. RNA extraction was performed using the RNeasy Plus Mini Kit (QIAGEN), followed by mRNA enrichment using rRNA depletion. RNA integrity was verified with the Experion RNA HighSens Analysis kit (Bio-Rad Laboratories), with samples meeting a minimum RNA Integrity Number and a 260/280 absorbance ratio for inclusion. Sequencing libraries were prepared using Illumina TruSeq Stranded mRNA reagents and sequenced on the NovaSeq 6000 platform (paired-end, 150 bp). The reads were mapped to the human reference genome GRCh37/hg19 using Ensembl gene models (v.92) and quantified with Kallisto (v.0.43.1)^1,21,123^. After quality control, transcript expression levels were calculated as transcripts per million, with further normalization performed using trimmed mean of *M*-values with NOISeq and batch effect correction applied using limma^124^. The resulting normalized transcripts per million were log_10_-transformed and mapped to the Destrieux Atlas^125^ based on region name matches.

### Cerebral blood flow data

Lastly, we performed a sanity check by replicating previously known associations between vasomodulatory neuropeptide receptors and cerebral blood flow. Cerebral blood flow data come from the HCP-Aging dataset^126^, estimated using ASL^127,128^. The acquisition is based on a pseudo-continuous ASL and two-dimensional multiband echo-planar imaging sequence^129^. Pseudo-continuous ASL data were acquired with labeling duration = 1,500 ms and five post-labeling delays = 200 ms, 700 ms, 1,200 ms, 1,700 ms and 2,200 ms, containing 6, 6, 6, 10 and 15 control-label image pairs, respectively. Other parameters related to this sequence include: spatial resolution = 2.5 × 2.5 × 2.5 mm^3^, repetition time/echo time = 3,580/18.7 ms. Two M0 images for blood perfusion quantification were acquired at the end of all the acquisitions. Preprocessing was done according to the HCP pipeline for ASL data (https://github.com/physimals/hcp-asl)^130–134^. The resulting cerebral blood flow maps from *n* = 678 individuals were then averaged into a group template and mapped to the Schaefer 400 cortical^17^ and Melbourne Subcortex S4 (ref. ^18^) atlas.

### Reporting summary

Further information on research design is available in the Nature Portfolio Reporting Summary linked to this article.

## Online content

Any methods, additional references, Nature Portfolio reporting summaries, source data, extended data, supplementary information, acknowledgements, peer review information; details of author contributions and competing interests; and statements of data and code availability are available at 10.1038/s41593-026-02236-w.

## Supplementary information

Supplementary Information Supplementary Table 1 and Figs. 1–6.

Reporting Summary

## Source data

Source Data Statistical source data.

## Data Availability

The preprocessed gene expression data used to perform the analyses can be found at https://osf.io/4rsz9. Raw gene expression data (bulk tissue microarray and RNA-seq) can be found in the AHBA: http://human.brain-map.org. Gene expression data (bulk RNA-seq) used for replication can be found in the Protein Atlas: https://www.proteinatlas.org. Orthologous AASs and coding sequences can be found in the NCBI database: https://www.ncbi.nlm.nih.gov. PET maps of opioid receptors can be obtained from neuromaps: https://github.com/netneurolab/neuromaps. The Neurosynth data are available at https://neurosynth.org. The HCP-Aging dataset, including Arterial Spin Labeling MRI, is available at https://db.humanconnectome.org. Source data are provided with this paper.

## Code availability

All code used to perform the analyses can be found at https://github.com/netneurolab/ceballos_neuropeptide-organization.
