# Supplementary material for: Organization of neuropeptide systems in the human brain
Source: Nat Neurosci. 2026 Mar 17;29(5):1212–24. doi: 10.1038/s41593-026-02236-w (PMC13156038; doi:10.1038/s41593-026-02236-w)
Supplement: Supplementary file 1 — Supplementary Table 1 and Figs. 1–6. [file 41593_2026_2236_MOESM1_ESM.pdf]

nature neuroscience

Supplementary information https://doi.org/10.1038/s41593-026-02236-w

Organization of neuropeptide systems in the human brain

In the format provided by the authors and unedited

	Species	**LRT** [min, max]	**dn/ds** [min, max]	$\mathbf{P_{FDR}}$ [min, max]	**P** [min, max]
ionotropic	pmarinus	[-0.0237, 0.0138]	[-0.2241, 2.1675]	[0.7787, 1]	[0.4792, 1]
	ccarcharias	[-0.0107, 4.6403]	[0, 1.3964]	[0.4644, 1]	[0.0357, 1]
	drerio	[-0.0120, 7.1551]	[-0.1111, 3.6990]	[0.0642, 1]	[0.0099, 1]
	xtropicalis	[-0.0201, 4.3531]	[-0.1739, 3.2348]	[0.2326, 1]	[0.0414, 1]
	ggallus	[-0.0209, 8.0459]	[-0.0427, 2.9204]	[0.0408, 1]	[0.0063, 1]
	oanatinus	[-0.9953, 4.5537]	[-0.1271, 25]	[0.2428, 1]	[0.0374, 1]
	sharrisii	[0, 9.5760]	[-0.1246, 25]	[0.0376, 1]	[0.0029, 1]
	dnovemcinctus	[-0.0069, 6.1576]	[0, 1.9812]	[0.2135, 1]	[0.0164, 1]
	btaurus	[0, 54.6920]	[-0.0690, 3.8238]	[0, 1]	[0, 1]
	mmusculus	[0, 0]	[-0.1065, 0]	[1, 1]	[1, 1]
	mmulatta	[0, 55.9213]	[0, 8.6645]	[0, 1]	[0, 1]
	ptroglodytes	[0, 264.3335]	[0, 3.2348]	[0, 1]	[0, 1]
	hsapiens	[0, 1.5214]	[0, 10]	[0.7242, 1]	[0.1837, 1]
metabotropic	pmarinus	[-0.2754, 0.0026]	[-0.2438, 25]	[0.8125, 1]	[0.4915, 1]
	ccarcharias	[-2.9925, 13.1814]	[0, 5.1618]	[0.0020, 1]	[0.0005, 1]
	drerio	[-1.8602, 8.8158]	[-0.1876, 25]	[0.0276, 1]	[0.0043, 1]
	xtropicalis	[-17.6931, 9.4776]	[-0.1318, 3.6990]	[0.0395, 1]	[0.0030, 1]
	ggallus	[-1.5271, 18.1816]	[-0.1305, 3.2351]	[0.0002, 1]	[0, 1]
	oanatinus	[-0.3890, 45.8363]	[-0.2477, 25]	[0, 1]	[0, 1]
	sharrisii	[-0.6721, 20.7441]	[-0.1180, 25]	[0.0001, 1]	[0, 1]
	dnovemcinctus	[-0.0033, 19.6190]	[-0.0937, 25]	[0.0001, 1]	[0, 1]
	btaurus	[0, 56.3281]	[-0.1684, 25]	[0, 1]	[0, 1]
	mmusculus	[0, 20.0457]	[-0.2428, 3.6989]	[0.0001, 1]	[0, 1]
	mmulatta	[0, 28.4537]	[0, 25]	[0, 1]	[0, 1]
	ptroglodytes	[0, 139.6956]	[0, 25]	[0, 1]	[0, 1]
	hsapiens	[0, 0.6318]	[0, 10]	[0.8125, 1]	[0.3041, 1]
peptide	pmarinus	[-0.7728, 0.1815]	[-0.3171, 25]	[0.7222, 1]	[0.4080, 1]
	ccarcharias	[-0.0228, 6.3180]	[-0.0719, 10]	[0.0492, 1]	[0.0151, 1]
	drerio	[-0.1692, 5.5278]	[-0.2617, 10]	[0.1321, 1]	[0.0227, 1]
	xtropicalis	[-1.4249, 8.9375]	[-0.2069, 25]	[0.0520, 1]	[0.0040, 1]
	ggallus	[-0.0005, 13.0529]	[-0.2268, 25]	[0.0065, 1]	[0.0005, 1]
	oanatinus	[0, 69.9689]	[-0.2289, 25]	[0, 1]	[0, 1]
	sharrisii	[-1.2041, 23.7114]	[-0.2955, 25]	[0, 1]	[0, 1]
	dnovemcinctus	[0, 21.4206]	[0, 3.8238]	[0.0001, 1]	[0, 1]
	btaurus	[-0.0379, 78.5394]	[0, 3.6990]	[0, 1]	[0, 1]
	mmusculus	[0, 5.2788]	[-0.1430, 3.8238]	[0.1673, 1]	[0.0257, 1]
	mmulatta	[0, 141.4610]	[0, 3.0433]	[0, 1]	[0, 1]
	ptroglodytes	[0, 214.3976]	[0, 25]	[0, 1]	[0, 1]
	hsapiens	[0, 2.0148]	[0, 10]	[0.5725, 1]	[0.1407, 1]

TABLE S1. **aBSREL results overview** | Exact values of all ranges of likelihood ratio tests (LRT) and substitution rates (dn/ds) for each branch of the aBSRSEL model, stratified according to receptor type. These data are displayed as boxplots in Fig 6.

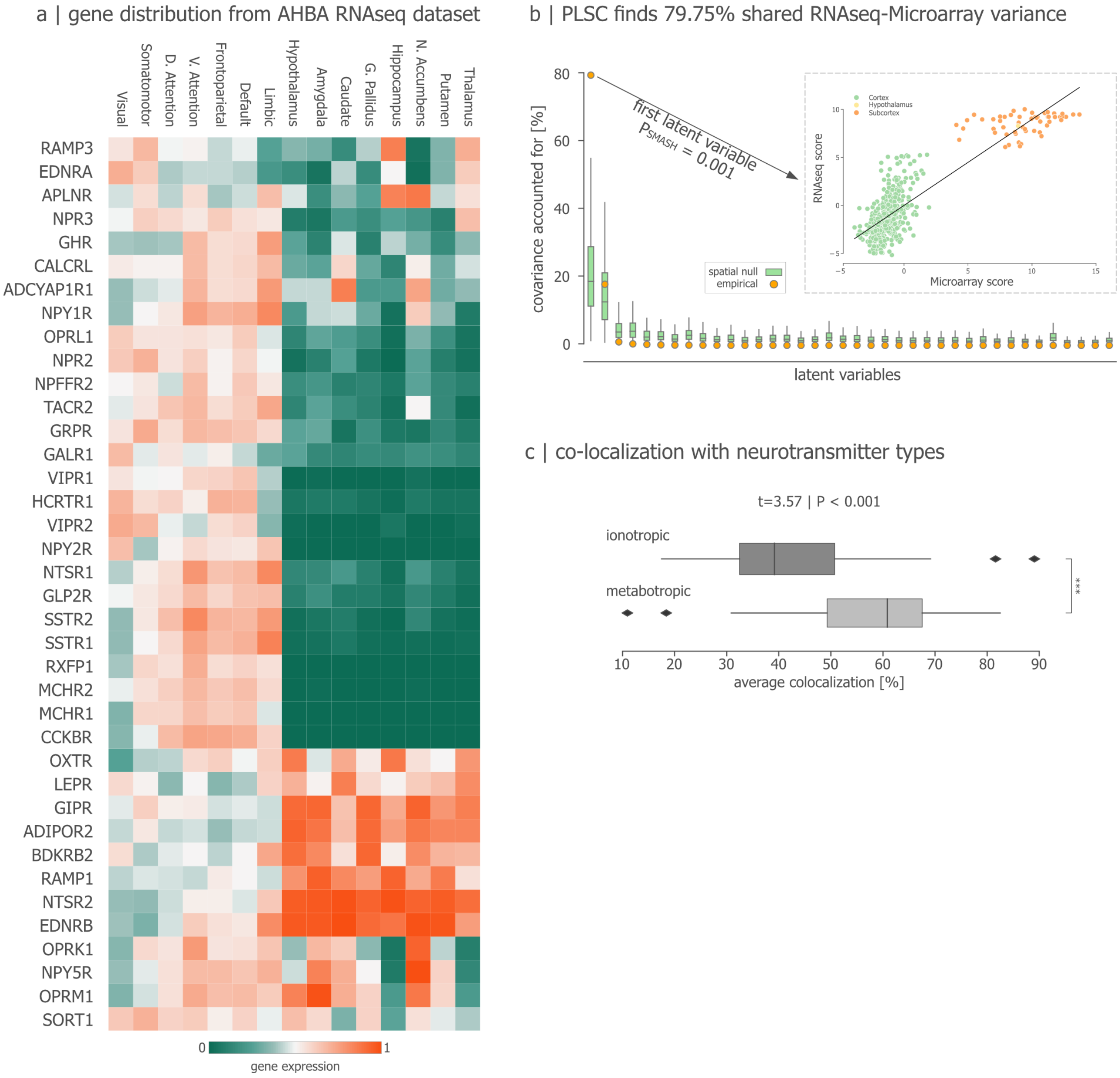

Figure S1. **Replication with RNA-seq data from the Allen Human Brain Atlas** | We replicated the main findings of our study using RNA-seq data available from two individuals in the AHBA. (a) We find, again, that neuropeptide receptors organize into predominantly cortical or subcortical. (b) The bulk microarray and RNA-seq measurements share around 80% of their variance when optimised with Partial Least Squares Correlation (PLSC). We find that the first latent variable found with PLSC is significantly higher than data spatially permuted with spatial autocorrelation-preserving randomization ($P_{\text{SMASH}} = 0.001$). (c) Co-localization to classical neurotransmitter systems remains significantly higher for metabotropic than ionotropic. Asterisks denote statistical significance under a two-tailed t-test ($t(74) = 3.57$, $P < 0.001$). Bounds of the boxplots in **b** and **c** represent the 1st (25%) and 3rd (75%) quartiles; the center line represents the median; whiskers represent the non-outlier endpoints of the distribution; and diamonds represent outliers.

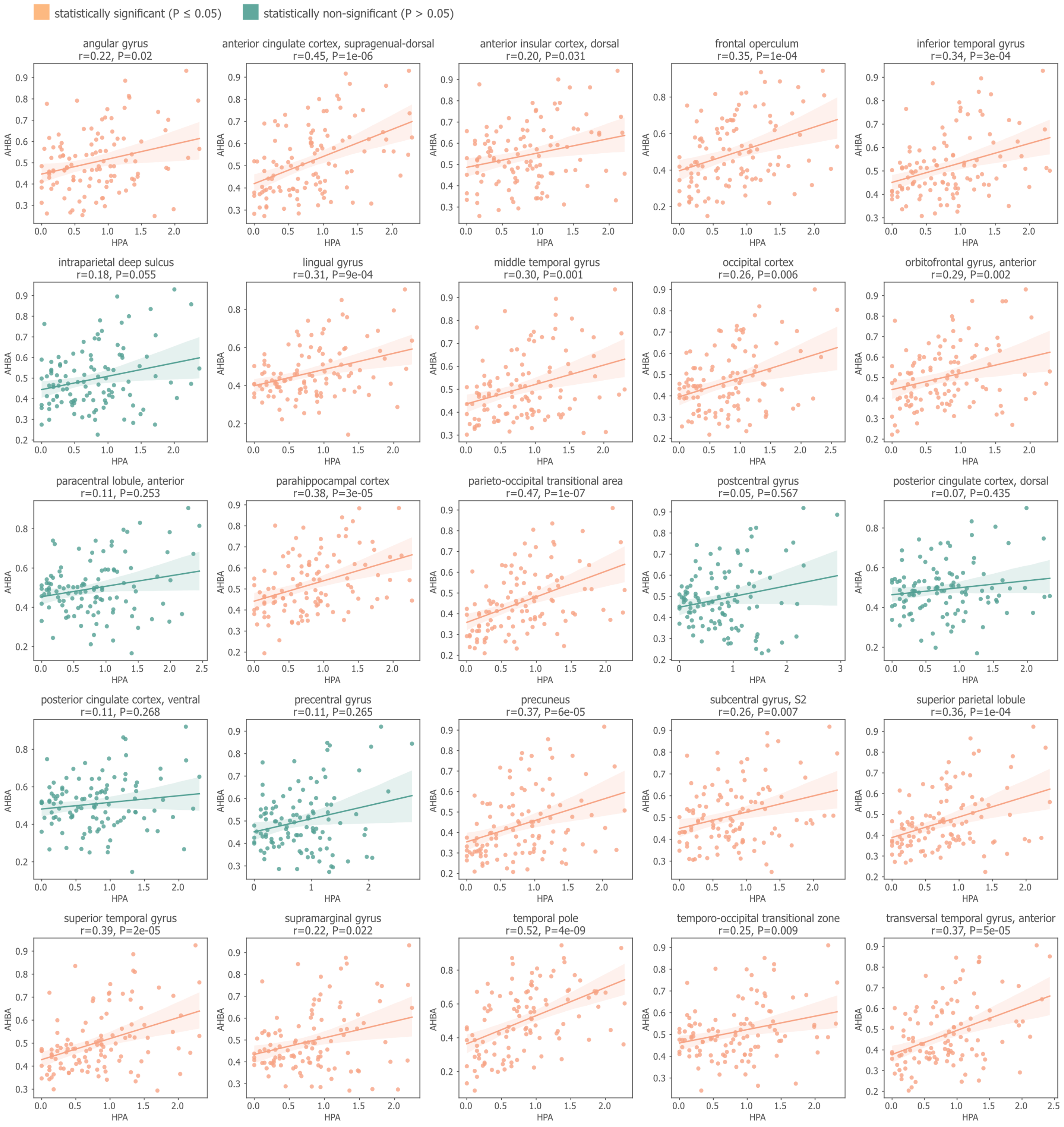

Figure S2. **Comparisons with RNA-seq data from the Human Protein Atlas (HPA)** | To assess the consistency of spatial patterns of gene expression in the discovery data set (microarray bulk sequencing from the Allen Human Brain Atlas) [16], we systematically compare it with RNA-seq data from the Human Protein Atlas (HPA) [20, 21]. The AHBA data were parcellated according to the Destrieux atlas [126], and matched to the samples in HPA [20, 21], resulting in $n = 25$ regions of interest. Each panel corresponds to an anatomical region, with expression of neuropeptide receptor genes shown as points. The x-axis shows gene expression in HPA and the y-axis shows gene expression in AHBA. Orange and green plots indicate regions where linear associations are statistically significant ($P \leq 0.05$) and non-significant ($P > 0.05$), respectively. We find positive correspondences between the datasets in all matched regions, and statistically significant correspondence in 19/25 regions. Shading around regression line represents 95% confidence interval from $n = 1000$ bootstrap permutations.

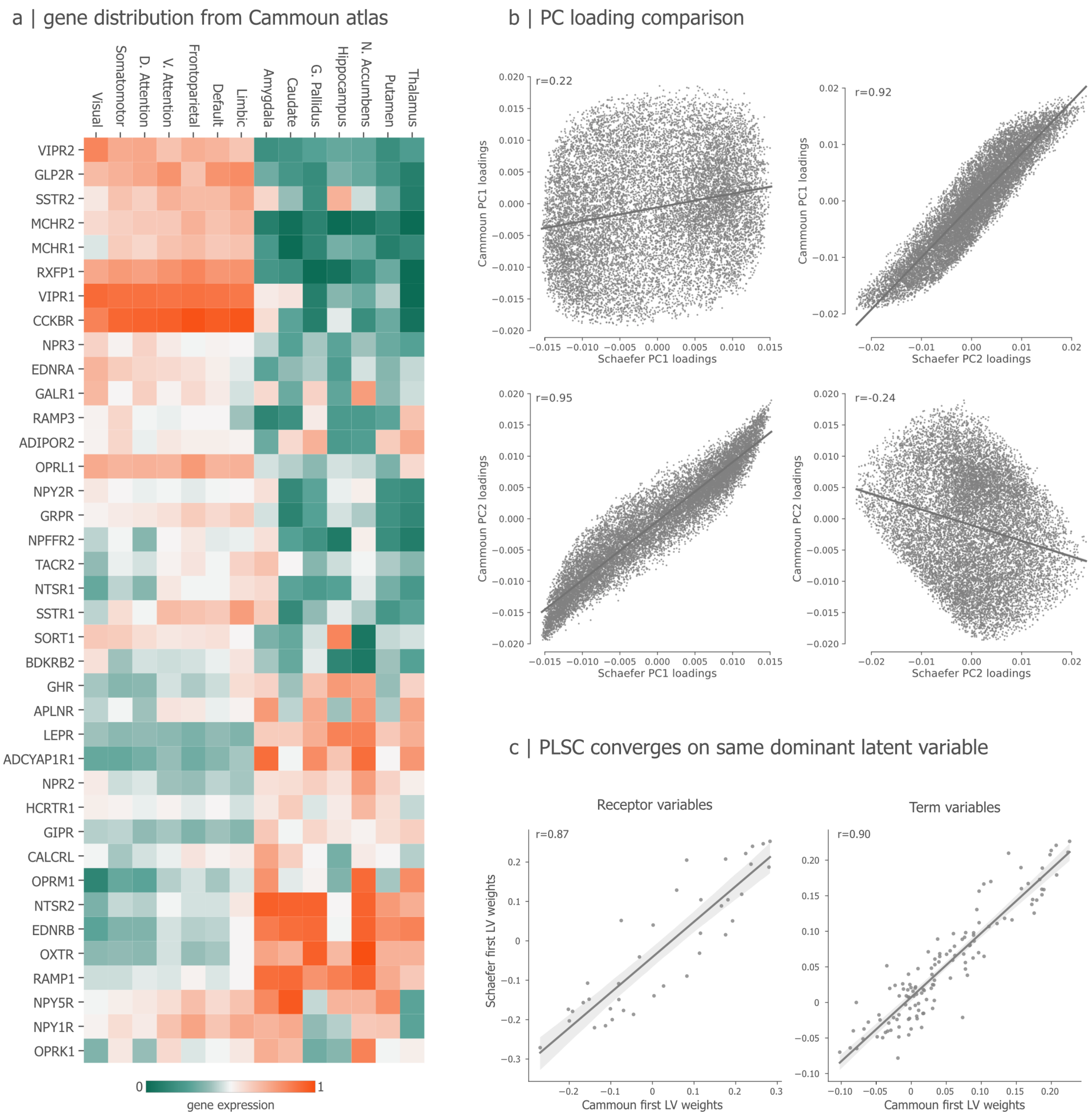

Figure S3. **Replication with an anatomical landmark-based atlas** | Main text figures are all shown for a functionally-derived atlas of the cortex and subcortex [17, 18]. Here we re-parcellate the cortical data according the Cammoun atlas [50], a subdivision of the landmark-based Desikan-Killiany atlas [132], yielding 500 regions of interest. We apply a corresponding FreeSurfer anatomical parcellation to the subcortex [133]. (a) Gene expression matrix of neuropeptide receptors, organized identically to Fig. 2. (b) PC1 and PC2 of the functional and anatomical parcellations. Collectively, the two PCs account for approximately equal portions of variance (PC1=16.58%, PC2=15.38%), but have reversed order. Namely, anatomical PC1 closely matches functional PC2 ($r = 0.92, p = 0$), and anatomical PC2 closely matches functional PC1 ($r = 0.95, p = 0$). (c) To ensure this reversal does not affect our covariance-based PLSC analysis in Fig. 5, we repeat the analysis using the anatomical parcellation. The scatterplots show positive correspondence between the PLSC weights (singular vectors) derived using the anatomical and functional parcellations (receptor weight similarity $r = 0.87$, $P = 10^{-12}$; term weight similarity $r = 0.9$, $P = 6 \times 10^{-47}$. Shading around regression lines in **c** represents 95% confidence interval from $n = 1000$ bootstrap permutations.)

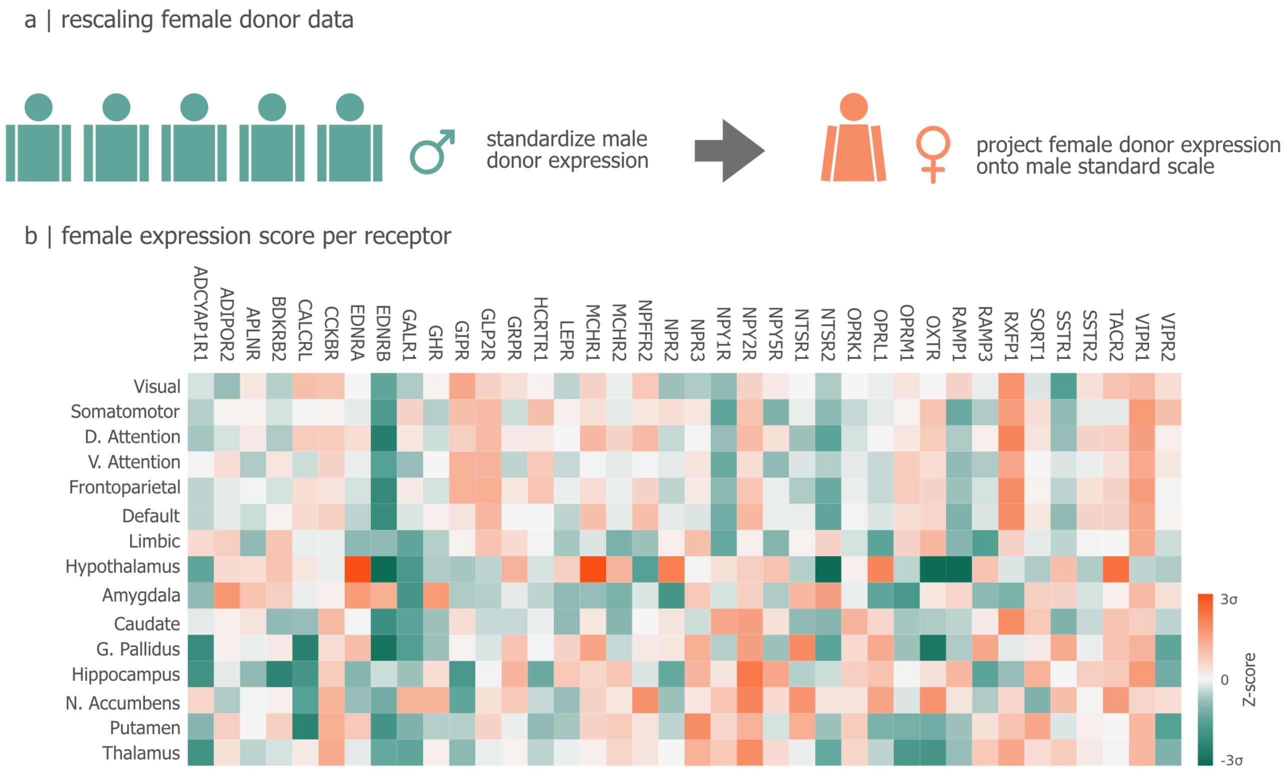

Figure S4. **Sex differences in neuropeptide receptor expression** | (a) To identify receptors with differential expression between the single female donor and the remaining five male donors, we z-score the female donor data with respect to male donor expression. (b) The resulting gene distribution highlights neuropeptide receptor genes with greater (orange) or lower (green) differential expression in the female donor.

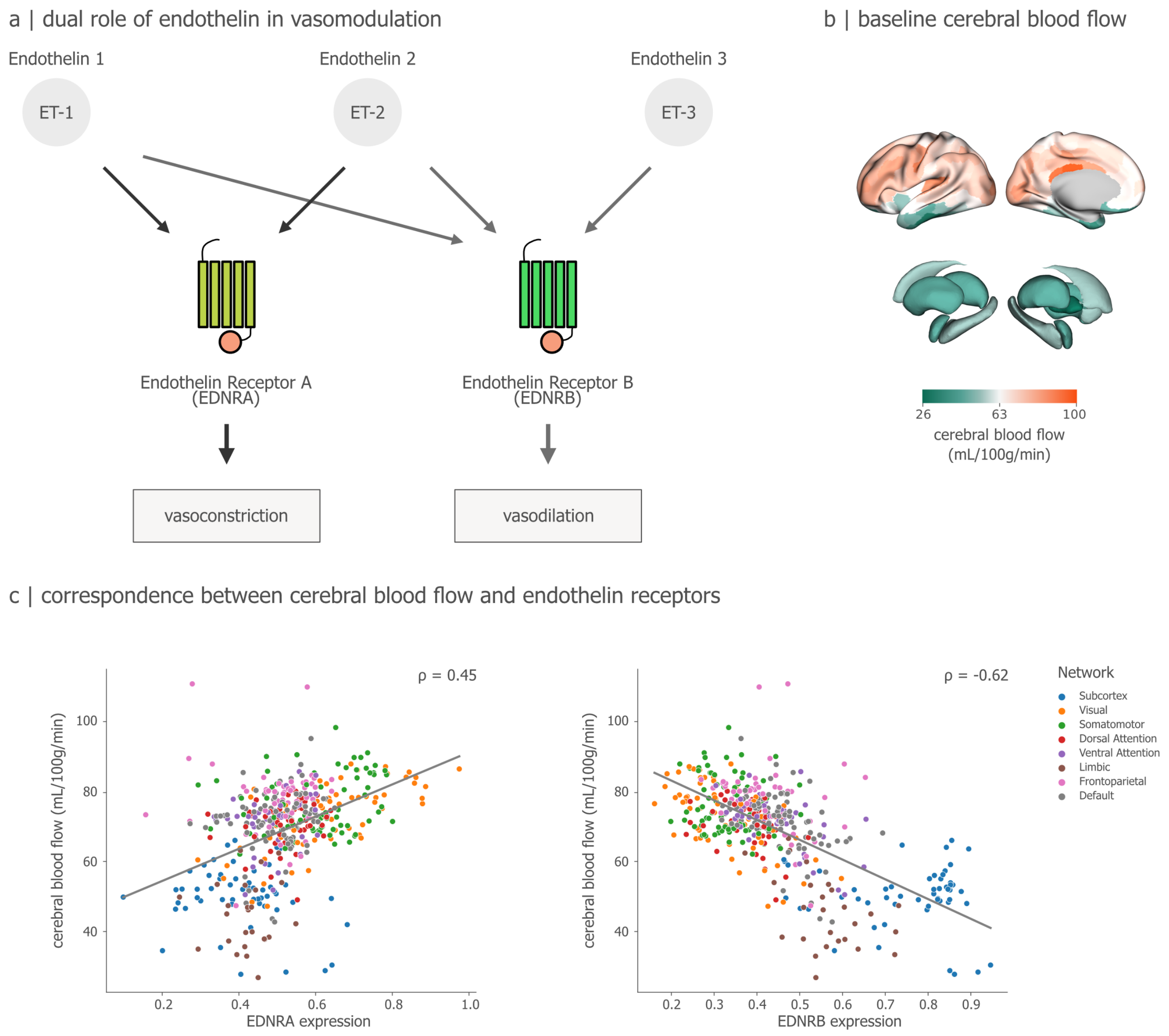

Figure S5. **Vasomodulatory neuropeptides correlate with cerebral blood flow** | To confirm specificity of neuropeptide mapping, we assess whether regional differences in the vasomodulatory endothelin receptors correspond to empirical measurements of cerebral blood flow, as measured using arterial spin labeling (ASL; see *Methods*). (a) We focus on two endothelin receptors, vasoconstrictive endothelin receptor A (EDNRA) and vasodilutive endothelin receptor B (EDNRB). (b) We use a group average template of cerebral blood flow from arterial spin labeling acquisitions of $n = 678$ individuals [127]. (c) Regional expression of vasoconstrictive EDNRA is positively correlated with cerebral blood flow ($\rho(452) = 0.45, P_{\text{SMASH}} = 0.0011$), while regional expression of vasodilutive EDNRB is negatively correlated with cerebral blood flow ($\rho(452) = -0.62, P_{\text{SMASH}} = 0.0001$). This is consistent with the regulatory role of endothelin peptides, responding to blood flow changes through extensive constriction in high perfusion and dilation in low perfusion areas, thereby keeping blood supply within physiological ranges [55, 56, 134, 135].

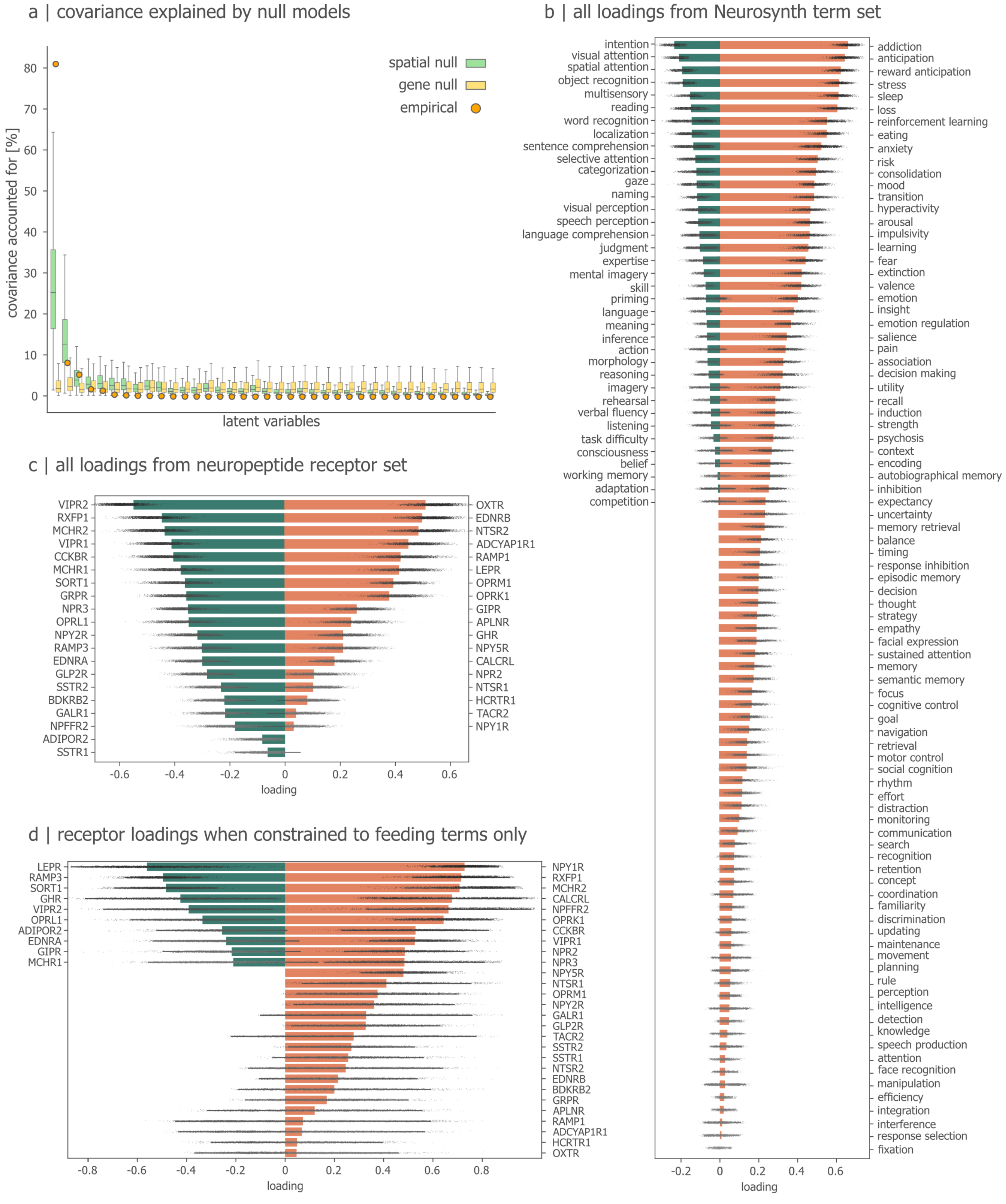

Figure S6. **Supporting information for the PLSC model** |(a) The covariance explained by each latent variable (orange dots) shown alongside null distributions generated using spatial autocorrelation-preserving randomization (green boxplots, each $n = 10000$) and matched null genes (yellow boxplots, each $n = 10000$) (see *Methods*). Boxplot bounds represents the 1st (25%) and 3rd (75%) quartiles; the center line represents the median; whiskers represent the endpoints of the distribution. (b) The full set of loadings for all Neurosynth terms in the Cognitive Atlas. (c) The full set of loadings for all neuropeptide receptors. (d) Loadings for neuropeptide receptors when the analysis is constrained only to feeding terms. Error bars in **b**, **c** and **d** represent bootstrap-estimated 95% confidence intervals from $n = 10000$ bootstrap permutations.
